# The RNA‐binding protein RBMS3 inhibits the progression of colon cancer by regulating the stability of LIMS1 mRNA

**DOI:** 10.1002/cam4.7129

**Published:** 2024-04-15

**Authors:** Yafei Li, Shuoshuo Wang, Guoli Li, Chunyang Gao, Zihan Cui, Mingqi Cong, Jie Hu, Minghui Zhang, Xiaoming Jin, Haiying Sun, Dan Kong

**Affiliations:** ^1^ Department of Pathology Harbin Medical University Harbin China; ^2^ Department of Anus and Intestine Surgery Chifeng Municipal Hospital Chifeng China; ^3^ Department of Anatomy Harbin Medical University Harbin China; ^4^ Central Operating Department The First Affiliated Hospital of Harbin Medical University Harbin China; ^5^ Department of Oncology Chifeng Municipal Hospital Chifeng China; ^6^ Gastrointestinal Rehabilitation Center Beijing Rehabilitation Hospital of Capital Medical University Beijing China; ^7^ Gastroenterology Department First Hospital of Dandong Dandong China; ^8^ Department of Gynaecology Tumor Hospital of Harbin Medical University Harbin China

**Keywords:** cell proliferation, colon cancer, LIMS1, metastasis, RBMS3

## Abstract

**Background:**

The RNA‐binding motif single‐stranded interacting protein 3 (RBMS3) is a constituent of the RNA‐binding motif (RBM) protein family, which assumes a pivotal role in governing cellular biogenesis processes such as the cell cycle and apoptosis. Despite an abundance of studies elucidating RBMS3's divergent roles in the genesis and advancement of various tumors, its involvement in colon cancer remains enigmatic.

**Methods:**

The present investigation employed data analysis from TCGA and GTEx to unveil that RBMS3 expression demonstrated a diminished presence in colon cancer tissues when juxtaposed with normal colon tissues. The effect of RBMS3 and LIM zinc finger domain 1 (LIMS1) on colon cancer was substantiated via animal models and cellular experiments. The connection between RBMS3 and LIM zinc finger domain 1 (LIMS1) was verified by molecular biology methods.

**Results:**

The study conclusively ascertained that augmenting RBMS3 expression quells the proliferation, migration, and invasion of colon cancer cells. Furthermore, the inquiry unveiled a plausible mechanism through which RBMS3 impacts the expression of LIMS1 by modulating its mRNA stability. The investigation ascertained that RBMS3 inhibits the progression of colon cancer by regulating LIMS1. The inhibitory function of LIMS1 and RBMS3 is closely intertwined in colon cancer, with knocking down LIMS1 being able to rescue the inhibitory effect of RBMS3 overexpression on the functionality of colon cancer cell

**Conclusions:**

The discernments delineate RBMS3 as a novel suppressor of cancer via LIMS1, thereby bestowing fresh therapeutic possibilities and illuminating the intricacies of colon cancer.

## Graphical abstract

RBMS3 enhances the mRNA stability of LIMS1, which, in turn, impedes the proliferation, migration, and invasion of colon cancer cells.

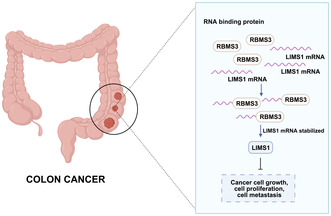

## 1 INTRODUCTION

Over recent years, the escalating incidence and mortality rates associated with colon cancer have emerged as a significant peril to human well‐being.^1^ Despite extensive research endeavors aimed at revealing the intricate molecular mechanism driving colon cancer, our understanding remains limited and necessitates further investigation. RNA‐binding motif (RBM) proteins, a cohort of RNA‐binding proteins, assume a vital role in governing vital RNA processes, including splicing, transport, translation, and stability.^2^ Numerous studies have highlighted the close association between abnormal expression and dysregulated function of RBM protein family members and the initiation and progression of various cancers. The RNA‐binding motif single‐stranded interacting protein 3 (RBMS3) gene, a constituent of the RBM protein family, is situated within the p23‐p24 region of human chromosome 3, with frequent observations of deletion or mutation in cancer, underscoring its pivotal contribution to cancer advancement.^3^ Multiple investigations have established RBMS3 as a tumor suppressor gene in cancer,^4^, ^5^ and its down‐regulation has been linked to unfavorable prognoses in malignancies such as esophageal squamous cell carcinoma, lung squamous cell carcinoma, nasopharyngeal carcinoma, and gastric cancer.^4^, ^6^, ^7^ However, RBMS3 has also been documented to exhibit promotive effects in breast cancer.^8^ The precise role of RBMS3 in colon cancer remains elusive, and its underlying molecular mechanisms remain incompletely elucidated. In this study, we demonstrate that RBMS3 exerts an inhibitory influence on colon cancer progression by impeding the malignant phenotype of colon cancer cells. Furthermore, our findings unveil LIM zinc finger domain 1 (LIMS1) as a pivotal gene mediating RBMS3's tumor‐suppressive effect. We establish that RBMS3 regulates the mRNA stability of LIMS1, consequently modulating LIMS1's expression level and impeding colon cancer progression.

## 2 MATERIALS AND METHODS

The materials and methods employed in this study were based on our previously published articles.^9^

### 2.1 The colon cancer data download and analysis

In this study, we performed a data analysis on RNA expression data of the RBM family gene in colon cancer. The dataset was obtained from the UCSC Xena database (https://xenabrowser.net/datapages), which included 639 samples collected from the TCGA and GTEx databases. The goal was to identify differential expression patterns between colon cancer tumors and normal colon tissues. To achieve this, we conducted cluster analysis and *t*‐test on the data with a significance level of *p* < 0.05. This allowed them to detect significant differences in gene expression between the tumor and normal samples. Furthermore, we obtained clinical staging data for colon cancer patients from the TCGA database. We utilized ANOVA (analysis of variance) to examine the variations in RBM family gene expression across different stages of colon cancer. ANOVA is a statistical test that assesses whether there are significant differences among multiple groups. The data analysis was performed using R 4.0.1. We also utilized GraphPad Prism 8.0 and the ComplexHeatmap R package to visualize and graphically represent the results of our analysis. These tools aided in the interpretation and presentation of the data. Overall, we aimed to characterize the expression patterns of RBM family genes in colon cancer, highlighting any significant differences between tumor and normal tissues, as well as exploring potential associations with different stages of the disease.

### 2.2 Collection of the colon cancer clinical samples

We meticulously accumulated a grand total of 20 specimens, encompassing both the pristine normal colon tissues as well as the afflicted colon cancer tissues, from the Tumor Hospital of Harbin Medical University. The vibrant tissues underwent a meticulous processing procedure, wherein they were deftly divided into two sections. One geometrically precise portion was expeditiously subjected to tantalizingly frigid liquid nitrogen, ensuring its instantaneous cryogenic preservation to facilitate the extraction of the precious RNA and protein molecules. Meanwhile, the other meticulously chosen segment was artfully submerged in a solution of 4% formaldehyde, inducing a state of graceful fixation, before being masterfully enveloped in a layer of refined paraffin, bestowing upon it an unparalleled elegance suitable for the ensuing immunohistochemistry (IHC) assay. The study protocol received approval from the Ethics Committee of Harbin Medical University, and informed consent was obtained from all participating patients.

### 2.3 IHC assay

We conducted an IHC assay to discern protein expression in human paraffin‐embedded colon cancer samples using anti‐RBMS3 (1:100, Abcam, Cambridge, UK, No. ab272612). The resultant IHC imagery was meticulously captured by the LEICA microscope system (German). Subsequently, two erudite pathologists independently evaluated the immunostaining outcomes based on the proportion of positive tumor cells as well as the intensity of the staining. The grading system employed entailed the following categorizations: 0 (indicating an absence of positive tumor cells); 1 (denoting 1%–25% positive tumor cells); 2 (corresponding to 25%–50% positive tumor cells); 3 (representing 50%–75% positive tumor cells) and 4 (signifying >75% positive tumor cells), alongside the respective staining intensity (−: 0; +: 1; + +: 2; +++: 3).

### 2.4 Cell culture

In this investigation, we employed three distinct cell lines, namely HCT15, HCT116, and 293T, sourced from the esteemed Cell Lines Service of Cellcook Biotech Co., Ltd., situated in Guangzhou, China. These cell lines have been duly certified by the provider, affirming their veracity and the absence of mycoplasma contamination, as validated through comprehensive short tandem repeat analysis. The utilization of the HCT116 and HCT15 cell lines enabled us to delve into the intricate role of RBMS3 and LIMS1, while lentiviral infection was skillfully executed using the 293T cells. The cultivation of HCT15 and 293T cell lines was meticulously undertaken in DMEM (HyClone, Logan, UT, USA), whereas the HCT116 cells thrived in 1640 medium (HyClone), both fortified with 10% Fetal bovine serum (FBS) procured from the esteemed Invitrogen in Carlsbad, California, USA. Additionally, the culture medium was supplemented with streptomycin at a concentration of 100 μg/mL and penicillin at a concentration of 100 IU/mL. The cells were incubated within a meticulously regulated environment, maintaining a temperature of 37°C within a humidified incubator characterized by a 5% CO_2_ concentration.

### 2.5 Cell transfection and lentiviral infection

For the construction of the RBMS3 and LIMS1 overexpression plasmid, we successfully integrated RBMS3 and LIMS1 genes into a pLVX‐Puro lentivirus expression vector (TaKaRa, OSA, Japan). Additionally, to facilitate the creation of shRNAs targeting RBMS3 or LIMS1, we synthesized and inserted them into a lentivirus expression vector PLKO.1 (TaKaRa). Subsequently, to generate lentivirus particles, the lentivirus vectors encompassing RBMS3, LIMS1, or the respective shRNAs were transfected into 293T cells, utilizing a package system involving additional plasmids: psPAX2 (Addgene) and PMD2.g (Addgene). The resultant lentivirus particles were then skillfully transduced into both HCT116 and HCT15 cells. To assess the effectiveness of our genetic manipulations, we meticulously evaluated the expression levels of RBMS3 and LIMS1 within the colon cancer cell lines. This comprehensive evaluation involved the deployment of Western blot analysis, enabling the visualization and quantification of the protein expression levels, as well as quantitative PCR (qPCR), enabling us to determine the corresponding mRNA expression levels accurately.

### 2.6 Plasmid construction and dual luciferase reporter assay

In this study, we constructed a plasmid that contained the amplified 3′UTR sequence of LIMS1 from genomic DNA, using the specified primers listed in Table S1. Additionally, we also included LIMS1 mutant variants in the plasmid construct. The amplified 3′UTR sequence of LIMS1, along with the mutant variants, was successfully inserted into the pGL3‐LUC reporter vector obtained from Promega, located in Madison, USA. This vector is a widely used tool for luciferase reporter assays, allowing the measurement of gene expression regulatory elements. In order to investigate the impact of RBMS3 binding sites on LIMS1, HCT116 cells were seeded into 24‐well plates. Subsequently, the cells were co‐transfected with the pGL3‐LUC reporter vector containing the 3′UTR sequences of LIMS1 (with wild‐type and mutant variants) and the PRL‐TK Renilla luciferase reporter vector, both obtained from Promega. The co‐transfection was achieved using Lipofectamine 3000 (Invitrogen, Carlsbad, CA, USA).To quantify the activities of Firefly and Renilla luciferase reporters, a Promega GloMax®20/20 Luminometer E5311 was employed. To ensure statistical accuracy and reliability, the quantification process was repeated three times.

### 2.7 MTT cell viability assay

In order to evaluate cell viability, we followed a defined protocol. Initially, we re‐suspended cells in the logarithmic growth phase at a concentration of 5 × 10^4^ cells/mL. Subsequently, 100 μL of the cell suspensions, corresponding to 5000 cells, were seeded into each well of a 96‐well plate and cultured for 24, 48, and 72 h in a 37°C incubator. After the specified incubation periods, 20 μL of MTT solution (5 mg/mL) was added to each well and allowed to incubate for 3 to 4 h. Following this, the previous culture medium was carefully aspirated, and 150 μL of DMSO solution was introduced into each well to solubilize the formazan crystals formed by the MTT. Subsequently, the entire 96‐well plate was placed in an incubator at 37°C for a duration of 10 min to ensure complete solubilization of the formazan crystals. Finally, the absorbance was measured at a wavelength of 490 nm using a microplate reader. To calculate the multiple of cell viability, the number of cells at different time points was divided by the number of cells at 0 h. This calculation allows for the assessment of the relative change in cell viability over the designated time intervals.

### 2.8 EdU (5‐ethynyl‐2 deoxyuridine) determination

In order to ascertain cell proliferation, the utilization of the EdU kit, fabricated by the esteemed Beyotime Institute of Biotechnology located in Shanghai, China was employed. The enthralling process involved the careful inoculation of HCT116 and HCT15 cells into 24‐well plates at a calculated density of 5 × 10^4^ cells per well, accompanied by a 24h period of meticulous cultivation. Thereafter, an introduction of 10 μM EdU eagerly took place within each well, allowing for an incubation duration of 2 h at a precisely maintained temperature of 37°C. Following this pivotal phase, a sublime fixation period of 15 min ensued, employing the ethereal essence of a 4% formaldehyde solution. To further illuminate the cellular landscape, a subsequent infiltration of 0.3% TritonX‐100 gently caressed the divine cells for a sublime span of 10 min. A symphony of scientific innovation continued with the introduction of a click reaction mixture of illustrious composition, entailing a volumetric serenade of 100 μL per well. This harmonious concoction was left to dance gracefully at a pleasing room temperature for a period of 30 min, allowing for the enchanting transformation of cellular matter. In order to bestow visual splendor upon the nuclei, a captivating staining ritual was performed utilizing the ethereal elixir known as Hoechst 33342. To complete this intriguing symphony of scientific exploration, the utopian landscape of cell proliferation was evaluated through the lens of an exquisite fluorescence microscope, artfully crafted by the craftsmen of Nikon in the resplendent nation of Japan. This arcane instrument unveiled the mesmerizing spectacle, enabling the determination of the percentage of cells that graciously displayed positive results.

### 2.9 Cell migration and invasion assay

The study encompassed an exploration of cell migration and invasion, which was meticulously executed employing a 24‐well Transwell chamber system, masterfully crafted by the esteemed Corning Incorporated (Corning, NY, USA). The experiment voyage commenced by suspending the cells in serum‐free DMEM, delicately nurturing them within the upper chamber at a density of 1 × 10^5^ cells, serenaded by a voluminous 400 μL. The lower chamber, on the other hand, was adorned with a nurturing culture media mixed generously with 20% FBS. Following a tranquil period of 24 h, the resilient cells were basked in the ethereal embrace of a 95% ethanol solution for a duration of 15 min to ensure their preservation. Thereafter, an enchanting performance of staining ensued, with the majestic 0.1% crystal violet, sourced gracefully from the renowned purveyor of scientific wonders, Sigma‐Aldrich, located in St. Louis, MO, USA. Carefully guided by a skilled hand, any cells that chose not to embark on the voyage of migration were compassionately ushered away from the upper compartment, their presence nullified by the gentle touch of a cotton swab. Subsequently, wielding the powers bestowed by a light microscope, three to five random areas were meticulously selected to bear witness to the captivating movements of both migratory and invasive cells, each deserving their respective moments in the spotlight. It is worth noting that prior to the enthralling inoculation of cells, the chambers of the invasion assay were lovingly coated with the sacred essence of Matrigel, a gift bestowed upon us by the venerable Sigma‐Aldrich.

### 2.10 The scratch assays

The scratch assay involved culturing colon cancer cells to confluency in a six‐well plate, creating a scratch in the cell monolayer, washing the cells with serum‐free culture medium, capturing initial images, incubating the cells in serum‐free culture medium, capturing subsequent images at specified time points (0, 24, 48 h), analyzing wound closure through image analysis, and quantifying and statistically analyzing the data to assess cell migration and wound healing.

### 2.11 Xenografted tumor models

In this study, female BALB/C nude mice aged 6–8 weeks were procured from the esteemed Vitalriver (Beijing, China). The mice were housed under SPF‐level conditions, ensuring a controlled and sterile environment for their well‐being. For the experimental design, three groups were established: the RBMS3 overexpression, RBMS3 knockdown, and the control group. Cells from each group were collected and resuspended in single‐cell suspension, utilizing PBS as the medium, at a density of 1 × 10^6^ cells per 50 μL. The tumor cells were then subcutaneously inoculated into the hind limbs of BALB/C nude mice, initiating the formation of subcutaneous tumors. The growth of this tumor was closely monitored and measured on the third day following inoculation. The mice were sacrificed when the tumor diameter reached approximately 1 cm. The subcutaneous tumors were surgically removed, meticulously photographed, weighed, and measured. To enable further experimentation, the tumors were embedded in paraffin.

Moreover, a caudal vein tumor metastasis model was established. The RBMS3 overexpression, RBMS3 knockdown, and control group cells were resuspended in a single‐cell suspension using PBS at a density of 1.5 × 10^6^ cells per 50 μL. These cells were individually injected into the BALB/C nude mice through the tail vein. After an observation period of 30 days, the mice were euthanized, and lung tissue was dissected to observe and photograph any lung tissue metastasis. Similarly, the lung tissues were embedded in paraffin for subsequent experiments. It is essential to highlight that all procedures involving animals in this study were reviewed and approved by the Animal Care Committee of Harbin Medical University, ensuring that the ethical considerations for the welfare and well‐being of the animals were met.

### 2.12 Hematoxylin and eosin **(**H&E) staining

In this study, a standard laboratory procedure called H&E staining was performed on 4.0 μm serial sections of the samples. To begin the staining process, the paraffin‐embedded tissue sections were deparaffinized, which involves removing the paraffin wax from the sections. Next, the sections were subjected to H&E‐stained, which involves the use of Mayer's hematoxylin solution (Solarbio, Beijing, China). The staining procedure was carried out according to the instructions provided by the manufacturer of the hematoxylin solution. Following the staining process, the microscope system from LEICA, a renowned German manufacturer, was utilized to capture images of the stained sections.

### 2.13 RNA sequencing (RNA‐seq) and data analysis

In the study, libraries were generated and sequencing was performed on the RBMS3 overexpressed cells, knockdown cells, and control cells. Paired‐end 150 bp sequencing runs were carried out on the NovaSeq6000 platform, provided by Annoroad genome (Beijing, China). Upon obtaining the raw sequencing data in FASTA format, the first step is to perform quality control on the raw data using FastQC (v0.11.9).^10^ Subsequently, Trimmomatic (v0.39)^11^ is utilized to remove adapter sequences from the raw data and filter out sequences of length 150 bp, resulting in clean data devoid of adapter contamination. Next, the clean data is aligned to the reference genome (Reference genome version: Homo_sapiens. GRCh38. primary_assembly) using STAR (v2.7.9a).^12^ Following the alignment, featureCounts (v2.0.1) is employed to annotate the alignment results using the annotation file version: Homo_sapiens.GRCh38.98, obtaining count values. The analysis is based on the original count values, with each sample containing two replicates. Subsequent differential analysis was conducted between the two groups using the DESeq2 R package (v3.16).^13^ The significance threshold for identifying differentially expressed genes was established as adjusted (adj.) *p*‐value <0.05 and Fold change (FC) > 1.5 or adj. *p*‐value <0.05 and FC < 0.75.

### 2.14 RNA extraction and quantitative real‐time polymerase chain reaction (qRT‐PCR)

The mRNA levels of RBMS3 and LIMS1 in colon cancer cell lines were measured using the qRT‐PCR. The process began with the extraction of total RNA from the colon cancer cell lines, which was achieved using the widely utilized TRIzol reagent (Invitrogen). Following RNA extraction, cDNA synthesis was carried out using PrimeScriptTM RT Master Mix (TaKaRa). For the qRT‐PCR analysis, the FastStart Universal SYBR Green Master from Yeasen Biotechnology situated in Shanghai, China, was used. This was performed on the LC96 real‐time PCR system manufactured by Roche (Switzerland). To ensure the accuracy and reliability of the data, GAPDH was employed as an internal control for standardization, and all PCR reactions were repeated three times to validate the results. Moreover, the comparison threshold method, represented by the formula 2^−ΔΔCt^, was utilized to normalize the repetitions. The primer sequences used for qRT‐PCR analysis were synthesized and provided by Genewiz. These primer sequences can be found in Table S1.

### 2.15 RNA immunoprecipitation (RIP)

In the RIP experiment, colon cancer cells in the logarithmic growth phase are lysed on ice, and the resultant cellular lysates undergo centrifugation to procure the supernatant. The lysis buffer comprised 1M HEPES, 1M NaCl, 0.5M EDTA (pH 8.0), 2.5M KCl, 1M MgCl2, NP‐40, glycerol, 1.5 mM DTT, 1 mM PMSF, and RNase inhibitor (10 U/mL). Protein A magnetic beads are then used to capture protein–RNA complexes, followed by overnight incubation with Flag‐Tag antibody (Cell Signaling Technology, Boston, MA, USA, No. 14793S) or IgG antibody (Cell Signaling Technology, No. 3900S) at 4°C. After magnetic separation and washing, RNA is extracted from the antibody‐protein‐RNA complexes using Trizol, and cDNA is synthesized via reverse transcription. Finally, the mRNA level of the target RNA, such as LIMS1, is quantified using qRT‐PCR.

### 2.16 mRNA stability assay

A mRNA stability was conducted on colon cancer cells (HCT116) in the logarithmic growth phase by culturing them in six‐well plates and exposing various groups of cells to 10 μg/mL of Actinomycin D (Selleck, Shanghai, China, No. S8964). Samples were gathered at different time intervals (0, 3, 6, and 12 h) and RNA was isolated for subsequent analyses. The impact of RBMS3 on the mRNA stability of LIMS1 was assessed using qRT‐PCR.

### 2.17 Western blot

Colon cancer cells were subjected to total protein extraction using RIPA buffer (Beyotime Institute of Biotechnology, Shanghai, China), and quantified utilizing a BCA kit (Beyotime Biotechnology Institute). The proteins were separated via SDS‐PAGE and transferred to a PVDF membrane (Millipore, MA, USA). The membrane was then incubated in 5% bovine serum albumin (BSA) for 2 h at room temperature. Immunoblotting was carried out employing a rabbit polyclonal antibody against RBMS3 (dilution 1:1000, Abcam, No. ab272612), a rabbit polyclonal antibody against LIMS1 (dilution 1:1000, Proteintech, Wuhan, China, No. 20772‐1‐AP), and a mouse monoclonal antibody against GAPDH (dilution 1:2000, Proteintech, Wuhan, China, No. 60004‐1‐Ig). The secondary antibody used in this study was procured from Santa Cruz Biotechnology, located in Dallas, TX, USA, and conjugated with horseradish peroxidase for detection. Color enhancement was achieved using ECL (Thermo Scientific).

### 2.18 Statistics

The mean ± standard deviation (SD) was used to present the data. Statistical analysis employed both a Student's *t*‐test or one‐way analysis of variance (ANOVA) utilizing GraphPad Prism 8.0 software (GraphPad Prism, CA, United States). The Metascape platform (https://metascape.org) was utilized for functional enrichment analysis of gene ontology (GO). Pearson's correlation analyses were undertaken using R packages. A *p* value <0.05 was considered statistically significant, while “ns” denoted “not significant.”

## 3 RESULTS

### 3.1 RBMS3 expression was significantly down‐regulated in colon cancer

**FIGURE 1:**
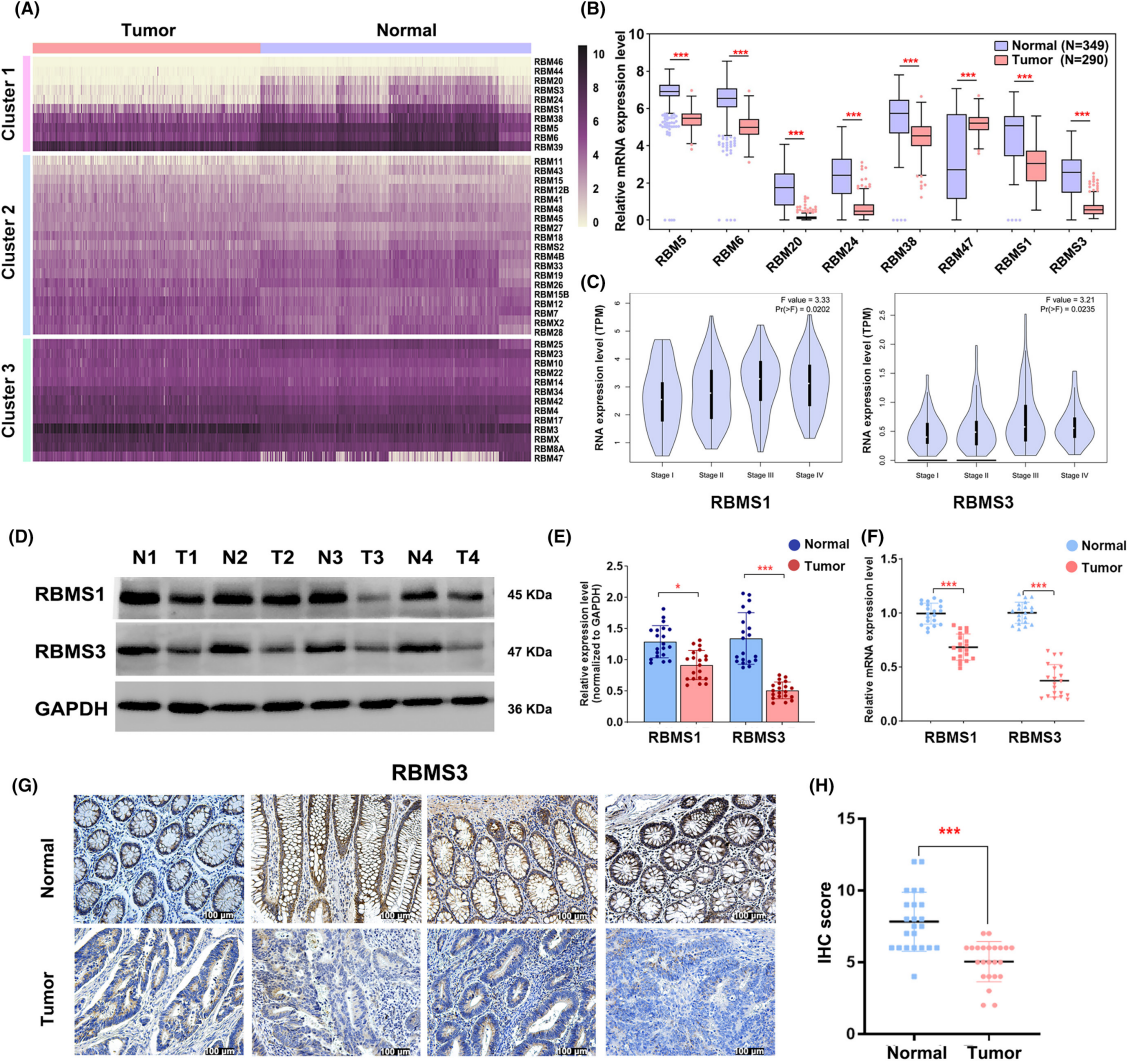
RBMS3 is down‐regulated in colon cancer tissues. (A) Cluster analysis of RBM family genes using data from TCGA and GTEx databases reveals three clusters. Genes in Cluster 1 are significantly downregulated in tumors, genes in Cluster 2 show no significant difference in expression between colon cancer and normal colon tissues, and genes in Cluster 3 are highly expressed in colon cancer tissues. (B) The expression levels of 8 RBM family genes in normal colon tissues and colon cancer tissues were analyzed using *t*‐tests. The results show a significant difference in expression between the two groups ***p < 0.001. (C) The correlation between RBMS1 and RBMS3 expression and clinical stage in colon cancer was analyzed using analysis of variance (ANOVA). A *p*‐value <0.05 indicates a significant correlation. (D) The expression levels of RBMS1 and RBMS3 in colon cancer tissues and normal colon tissues were detected using Western blot analysis. Representative images are shown here. A total of 20 pairs of tissues were tested. (E) The grayscale values of Western blot strips from 20 paired colon cancer and normal colon tissues were statistically analyzed using *t*‐tests, *p < 0.05, ***p < 0.001. (F) qRT‐PCR analysis was performed to evaluate the expression of RBMS1 and RBMS3 in colon cancer tissues and normal colon tissues, ***p < 0.001. (G, H) IHC analysis was conducted to examine the expression of RBMS3 in colon cancer tissues and normal colon tissues. Statistical analysis was conducted using *t*‐tests to assess the IHC scores. ***p < 0.001.

We conducted an analysis of the TCGA and GTEx databases to compare the expression of RBM family genes in colon cancer and normal colon tissues. The analysis delineated these genes into three distinct categories. The first cluster encompasses genes significantly downregulated in normal colonic tissues, including RBM5, RBM6, RBM20, RBM24, RBM38, RBM47, RBMS1, and RBMS3. The second cluster comprises genes with no significant differences in expression between colon cancer and normal colon tissues, such as RBM11, RBM43, etc. The third cluster comprises genes that are significantly upregulated in colonic cancer tissues, including RBM47, RBMX, etc. (Figure 1A,B). Further investigation revealed a significant correlation between the expression of RBMS1 and RBMS3 and the clinical stage of colon cancer (Figure 1C). Furthermore, we examined the expression of RBMS1 and RBMS3 in normal colon tissues and colon cancer using Western blot and qRT‐PCR. Our results unveiled a noteworthy reduction in the expression of RBMS1 and RBMS3 in colon cancer in comparison to normal colon tissues (Figure 1D–F, Figure S1A). Moreover, we determined the expression of RBMS3 in paired colon cancer and adjacent normal tissues through IHC, revealing a marked decrease in the expression of RBMS3 in colon cancer tissues (Figure 1G,H). These discoveries indicate the potential pivotal role of RBMS1 and RBMS3 in the context of colon cancer.

### 3.2 RBMS3 inhibits colon cell proliferation

**FIGURE 2:**
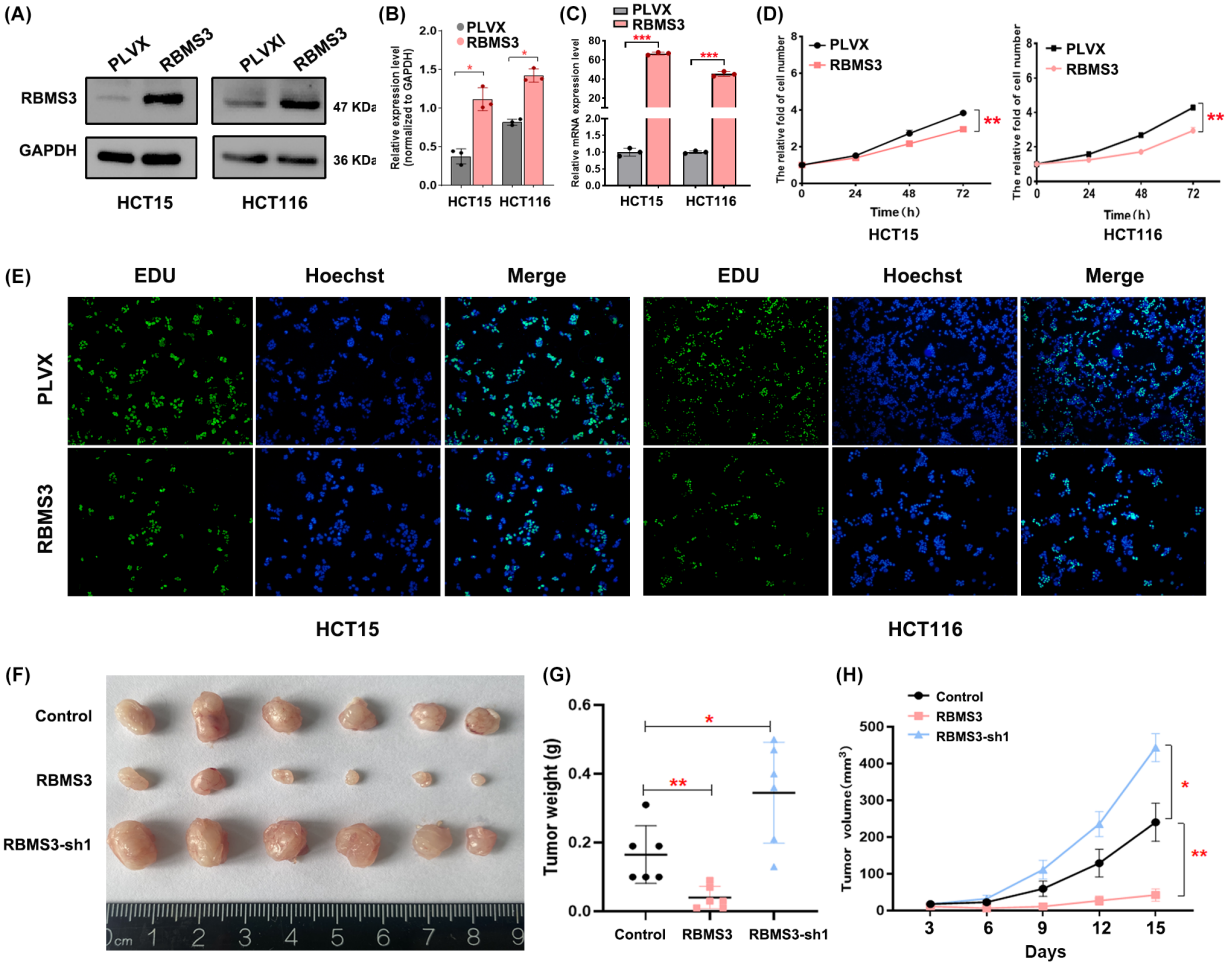
Overexpression of RBMS3 inhibits colon cancer proliferation. (A) Western blot analysis was conducted to evaluate the expression level of RBMS3 in both PLVX and RBMS3 overexpression cell models, PLVX was used as the control group. (B) The grayscale intensities of the Western blot bands were statistically analyzed, *p < 0.05. (C) RBMS3 expression was examined through qRT‐PCR in the PLVX control group and the group with RBMS3 overexpression, ***p < 0.001. (D) Cell proliferation was assessed using the MTT assay in both the RBMS3 overexpression group and the PLVX control group, **p < 0.01. (E) Cell proliferation was analyzed using the EDU assay in the RBMS3 overexpression group and the PLVX control group. (F) An experimental study employing a subcutaneous murine model was conducted to assess the growth condition of HCT116 cells inoculated with overexpressed RBMS3, knocked‐down RBMS3, and the control. Six mice were used in each group for the analysis. (G) Tumor weight was compared among the control group, RBMS3 group, and RBMS3‐sh1 group, *p < 0.05, **p < 0.01. (H) The tumor growth curve over time was plotted for the control, RBMS3, and RBMS3‐sh1 groups, *p < 0.05, **p < 0.01.

Our investigation primarily centered on elucidating the impact of RBMS3 on the proliferation of colon cancer cells.^14^, ^15^ To accomplish this, we established colon cancer cell lines (HCT116 and HCT15) with RBMS3 overexpressed and knockdown, which were subsequently confirmed through Western blot and qRT‐PCR (Figure 2A–C, Figure S1B–D). Utilizing MTT and EDU assays, we observed that overexpressing RBMS3 hindered the proliferation of colon cancer cells, whereas knockdown of RBMS3 promoted it (Figure 2D,E, Figure S1E–H). In vivo experiments further substantiated these findings, revealing that RBMS3 overexpression suppressed tumor cell growth, while its knockdown facilitated it (Figure 2F–H, Figure S1I–K). Hence, these outcomes signify the significant involvement of RBMS3 in regulation of colon cancer cell proliferation.

### 3.3 RBMS3 inhibits colon cancer cell metastasis

**FIGURE 3:**
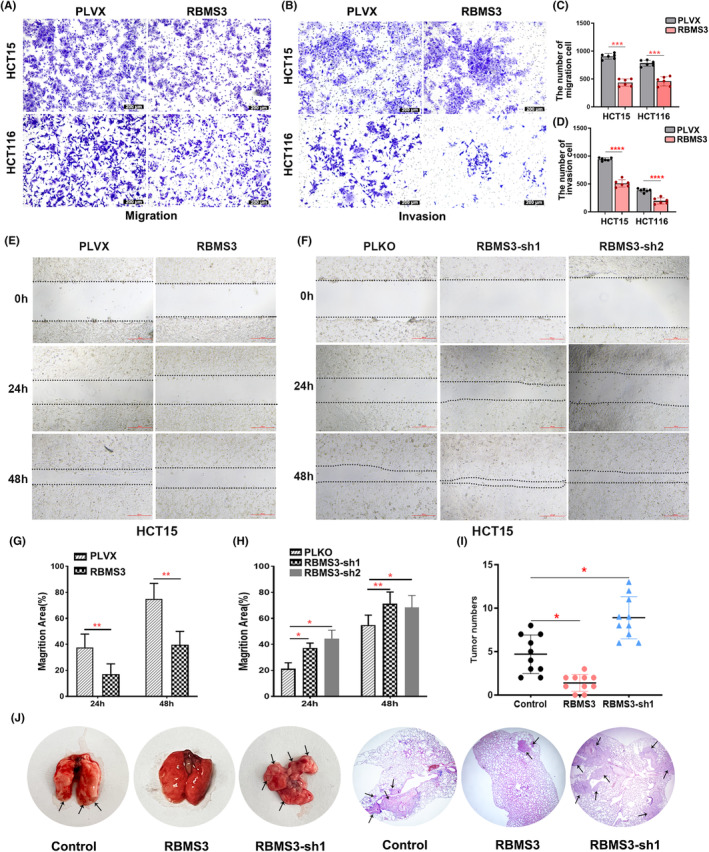
RBMS3 suppresses migration and invasion of colon cancer cells. (A, B) The transwell migration and invasion assays revealed that the overexpression of RBMS3 exerts an inhibitory effect on the migration and invasion of HCT15 and HCT116 cells (magnification, 100×; scale bars, 200 μm). (C, D) Statistical analysis of the cell migration and invasion among different groups was conducted using the *t*‐test, ***p < 0.001, ****p < 0.0001. (E, F) The scratch assay was employed to assess the migration of HCT15 cells in the overexpressed RBMS3, knocked‐down RBMS3, and control groups. (G, H) Statistical analysis was performed to evaluate the migration area of HCT15 cells in the overexpressed RBMS3, knocked‐down RBMS3, and control groups at different time points, *p < 0.05, **p < 0.01. (I) The number of lung metastatic lesions observed under the microscope in the overexpressed RBMS3, knocked‐down RBMS3, and control groups in the tail vein lung metastasis model were statistically analyzed using the *t*‐test, *p < 0.05. (J) The tail vein injection model was used to examine the influence of RBMS3 on the lung colonization of HCT116 cells in vivo, with a total of ten mice in each group. (magnification, 20×; scale bars, 500 μm; the black arrow represents the location of the metastasis).

Furthermore, we conducted an exploration into the influence of RBMS3 on the migration and invasion of colon cancer cells using transwell assays, scratch assays, and mouse models of lung metastasis. Our results consistently demonstrated that the overexpression of RBMS3 impeded the migration and invasion of colon cancer cells, while its knockdown facilitated these processes (Figure 3A–D, Figure S2). Notably, the scratch assay yielded similar results to the transwell migration and invasion assays (Figure 3E–H). Additionally, our in vivo experiments provided further evidence by showing that the overexpression of RBMS3 inhibited the lung colonization capacities of tumor cells, while the knockdown of RBMS3 promoted it (Figure 3I,J). These findings further support the role of RBMS3 in regulating the migration and invasion of colon cancer cells and suggest its involvement in suppressing metastasis.

### 3.4 LIMS1 is the critical target gene adjusted by RBMS3 in colon cancer

**FIGURE 4:**
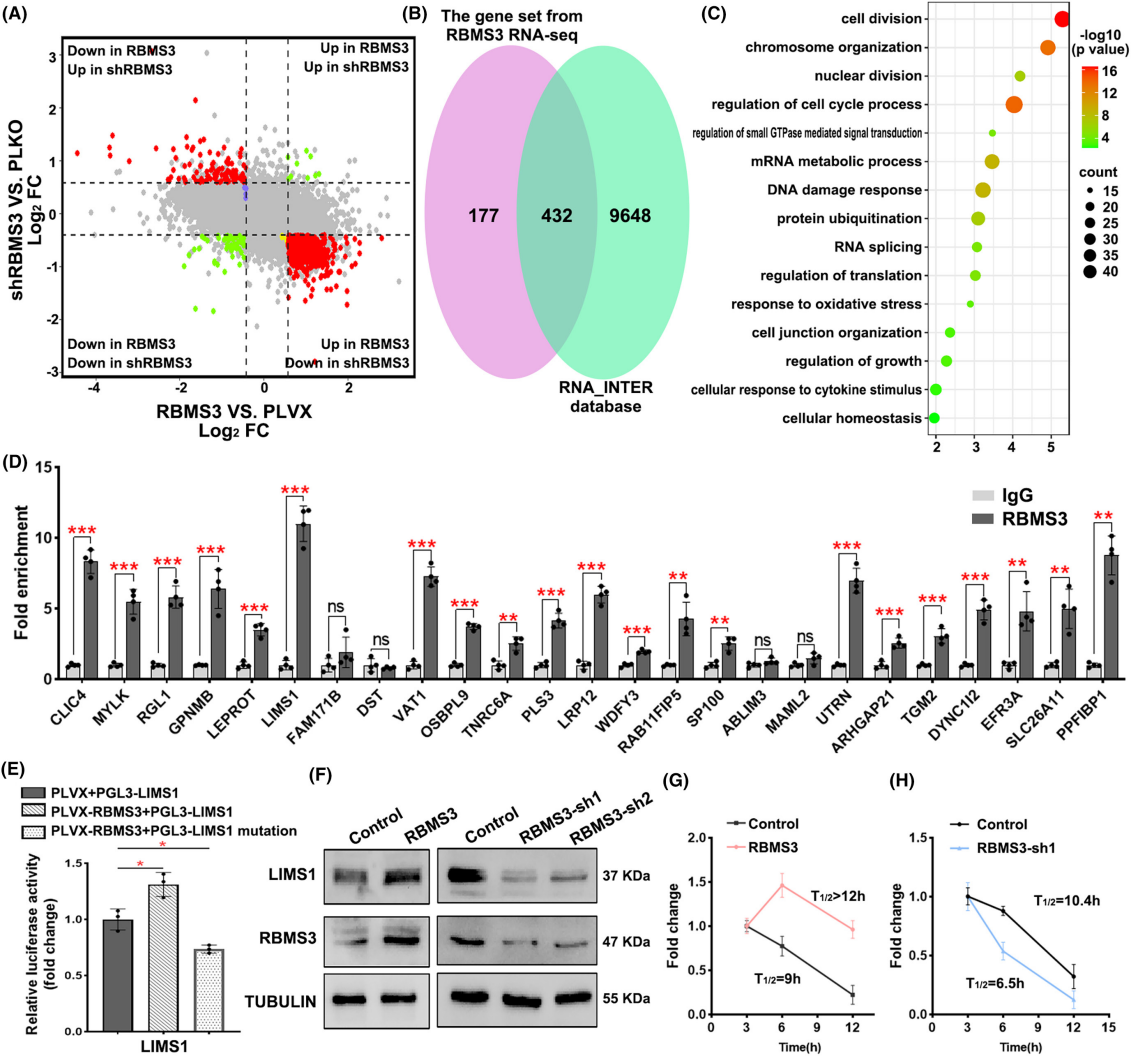
LIMS1 is the key target gene regulated by RBMS3. (A) Differential expression analysis was performed on the RNA‐seq data comparing the overexpression RBMS3 HCT15 cells and the control cells (pLVX group), as well as the shRBMS3 HCT15 cells and the control cells (PLKO group). The overexpression RBMS3 group showed 2576 upregulated genes and 3796 downregulated genes, while the shRBMS3 group exhibited 872 downregulated genes and 184 upregulated genes (cutoff: FC > 1.5 or FC < 0.75, *p*adj < 0.05). (B) In the 506 genes that were upregulated in the overexpression of RBMS3 and downregulated in the knockdown of RBMS3, and in the 103 genes that were downregulated in the overexpression of RBMS3 and upregulated in the knockdown of RBMS3, intersection analysis was performed with the predicted RBMS3 binding genes in the RNA‐INTER database, resulting in 432 candidate genes. (C) A GO enrichment analysis of the 432 candidate genes was conducted using the Metascape database. (D) A RIP assay was performed to measure the enrichment fold of RBMS3 binding target genes using HCT15 cells, *p < 0.05, **p < 0.01, ***p < 0.001, ns, not significant. (E) Dual luciferase assays confirmed the regulatory effect of RBMS3 on LIMS1 using HCT15 cells, *p < 0.05. (F) Western blot analysis was performed to assess the expression of LIMS1 in the control group and RBMS3 HCT15 cells, demonstrating increased LIMS1 expression with RBMS3 overexpression and decreased expression with shRBMS3 HCT15 cells (knockdown of RBMS3). (G) Treatment with Actinomycin D for 12 h revealed enhanced mRNA stability of LIMS1 in the RBMS3 overexpression group compared to the control group of HCT116 cells. (H) The stability of LIMS1 mRNA in the HCT116 cell line decreased upon RBMS3 knockdown, as observed after a 12‐h treatment with actinomycin D.

In order to elucidate the molecular mechanism underlying RBMS3's inhibitory effects on colon cancer progression, we performed RNA‐seq analyses on cells overexpressing RBMS3, cells with RBMS3 knocked down, and a control group. When comparing the overexpression group to the control group, we observed 2576 upregulated genes and 3796 downregulated genes. Conversely, when comparing the knockdown group to the control group, we found 872 downregulated genes and 184 upregulated genes (Figure 4A, Tables S2 and S3). To further investigate the genes influenced by RBMS3 expression, we conducted a meticulous analysis of the overlapping gene sets derived from profoundly upregulated or downregulated genes observed in the overexpression group (Set A) and the knockdown group (Set B). Consequently, this comprehensive examination culminated in the identification of 609 genes (Set C) whose expression levels were notably impacted by alterations in RBMS3 expression. Subsequently, to augment our investigation, we employed the RNA_Inter database to prognosticate RBMS3 binding mRNA. We then performed an astute assessment, evaluating the overlapping presence of the 432 genes with the anticipated RBMS3 target sites. Remarkably, this meticulous analysis unveiled the discovery of 432 prospective target genes, intricately associated with cell division, chromosome organization, nuclear division, mRNA metabolic process, regulation of cell cycle process, and cell junction (Figure 4B,C, Table S4). To further elucidate the pivotal target genes associated with the role of RBMS3, we conducted analyses using the TCGA and DepMap databases to explore the correlation between RBMS3 and the 432 potential target genes. The results revealed a positive correlation between RBMS3 mRNA levels and the mRNA levels of 83 target genes (Figure S3A, Table S5). Similarly, a positive correlation was observed between RBMS3 protein levels and the protein levels of 40 target genes (Figure S3B, Table S5). Notably, 25 genes exhibited consistent positive correlations with RBMS3 at both the mRNA and protein levels, suggesting that these 25 genes may represent critical target genes influenced by RBMS3 (Figure S3C, Table S5). Employing a RIP assay, we corroborated LIMS1 as a pivotal target gene of RBMS3 (Figure 4D). Additionally, the dual‐luciferase reporter assay elucidated the modulation of LIMS1 fluorescence activity by RBMS3. The assay demonstrated that RBMS3 heightened the activity of wild‐type LIMS1, while leaving mutant‐type LIMS1 unaffected (Figure 4E). Furthermore, Western blot analysis unveiled an increase in LIMS1 expression within RBMS3 overexpression group, correspondingly with the down‐regulation of RBMS3 expression. Concomitantly, the protein expression level of LIMS1 also exhibited a decrease (Figure 4F, Figure S3D). Lastly, we assessed the impact of altered RBMS3 expression on LIMS1 mRNA stability in colon cancer cells treated with Actinomycin D, revealing that RBMS3 amplified the stability of LIMS1 mRNA (Figure 4G,H). Overall, these findings strongly indicate that RBMS3 impedes the progression of colon cancer by governing the stability of LIMS1.

### 3.5 LIMS1 can inhibit colon cancer cell proliferation, migration, and invasion

**FIGURE 5:**
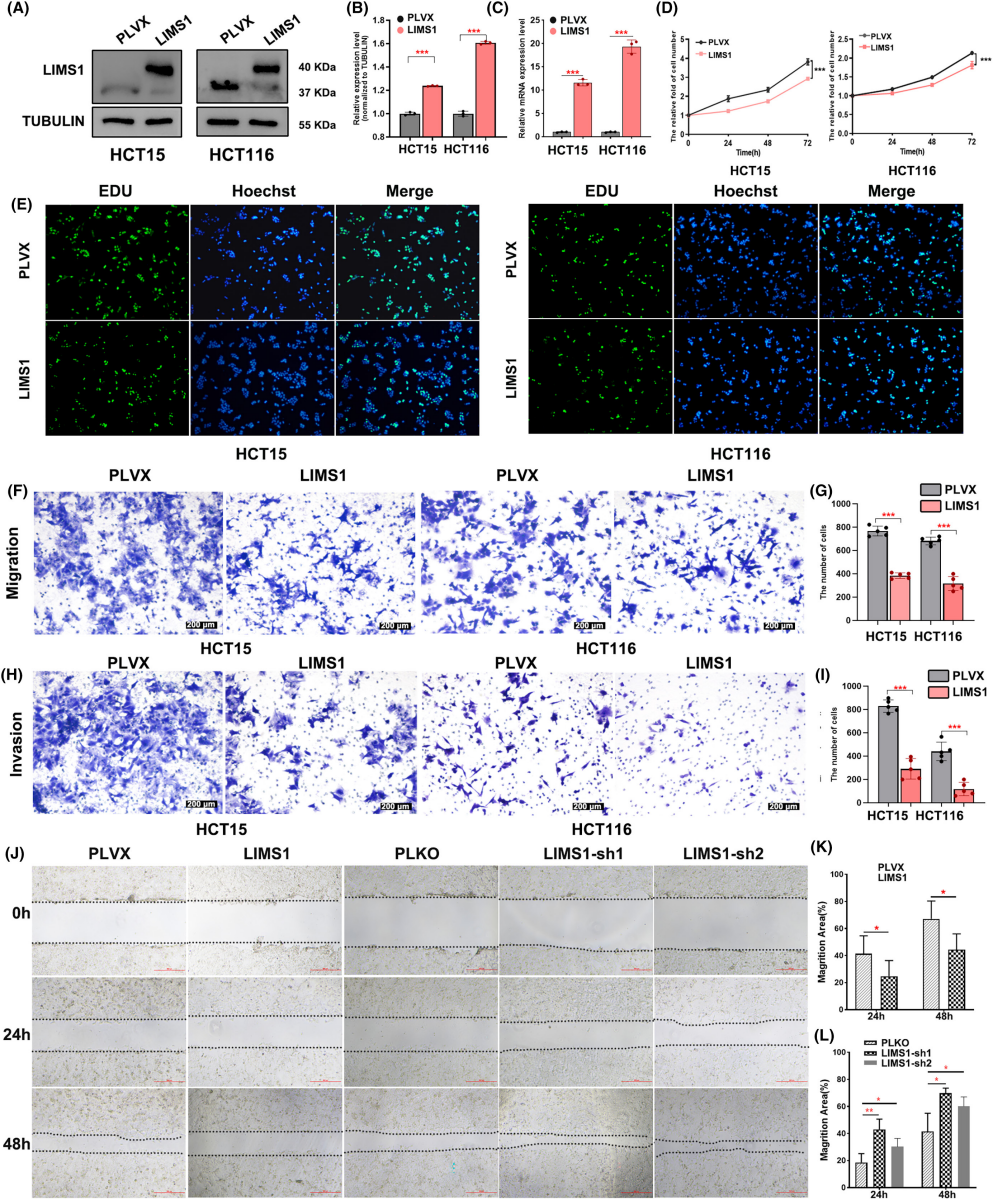
Overexpression of LIMS1 inhibits cell proliferation, migration, and invasion in colon cancer. (A) Western blot analysis was conducted to evaluate the expression level of LIMS1 in both PLVX and LIMS1 overexpression cell models, PLVX was used as the control group. (B) The grayscale intensities of the Western blot bands were statistically analyzed, ***p < 0.001. (C) LIMS1 expression was examined through qRT‐PCR in the PLVX control group and the group with LIMS1 overexpression, ***p < 0.001. (D) Cell proliferation was assessed using the MTT assay in both the LIMS1 overexpression group and the PLVX control group, ***p < 0.001. (E) Cell proliferation was analyzed using the EDU assay in the LIMS1 overexpression group and the PLVX control group. (F–I) The transwell migration and invasion assays revealed that the overexpression of LIMS1 exerts an inhibitory effect on the migration and invasion of HCT15 and HCT116 cells (magnification, 100×; scale bars, 200 μm). Statistical analysis of the cell migration among different groups was conducted using the *t*‐test, *p < 0.05, **p < 0.01. (J–L) The scratch assay was employed to assess the migration of HCT15 cells in the overexpressed LIMS1, knocked‐down LIMS1, and control groups. Statistical analysis was performed to evaluate the migration area of cells in the overexpressed LIMS1, knocked‐down LIMS1, and control groups at different time points, *p < 0.05, **p < 0.01.

In order to gauge the impact of LIMS1 on colon cancer cells, we established overexpressed and knockdown cell lines for LIMS1 in HCT116 and HCT15. The successful generation of LIMS1‐overexpressing and knockdown cells was validated through meticulous analysis via Western blot and qRT‐PCR analysis (Figure 5A–C and Figure S4A–C). Subsequently, we conducted MTT, EDU, Transwell assays, and scratch assays to assess the influence of LIMS1 on cell proliferation, migration, and invasion in colon cancer cells. Our investigations uncovered that the expression of LIMS1 significantly hindered the proliferation of colon cancer cells, while its knockdown notably accelerated proliferation (Figure 5D,E and Figure S4D–G). Furthermore, the results from the Transwell assay and scratch assay showed the expression of LIMS1 reduced the migration and invasion of colon cancer cells (Figures 5F–K and Figure S4H–K).

### 3.6 Knockdown LIMS1 can rescue the inhibiting effect of RBMS3 in colon cancer cells

**FIGURE 6:**
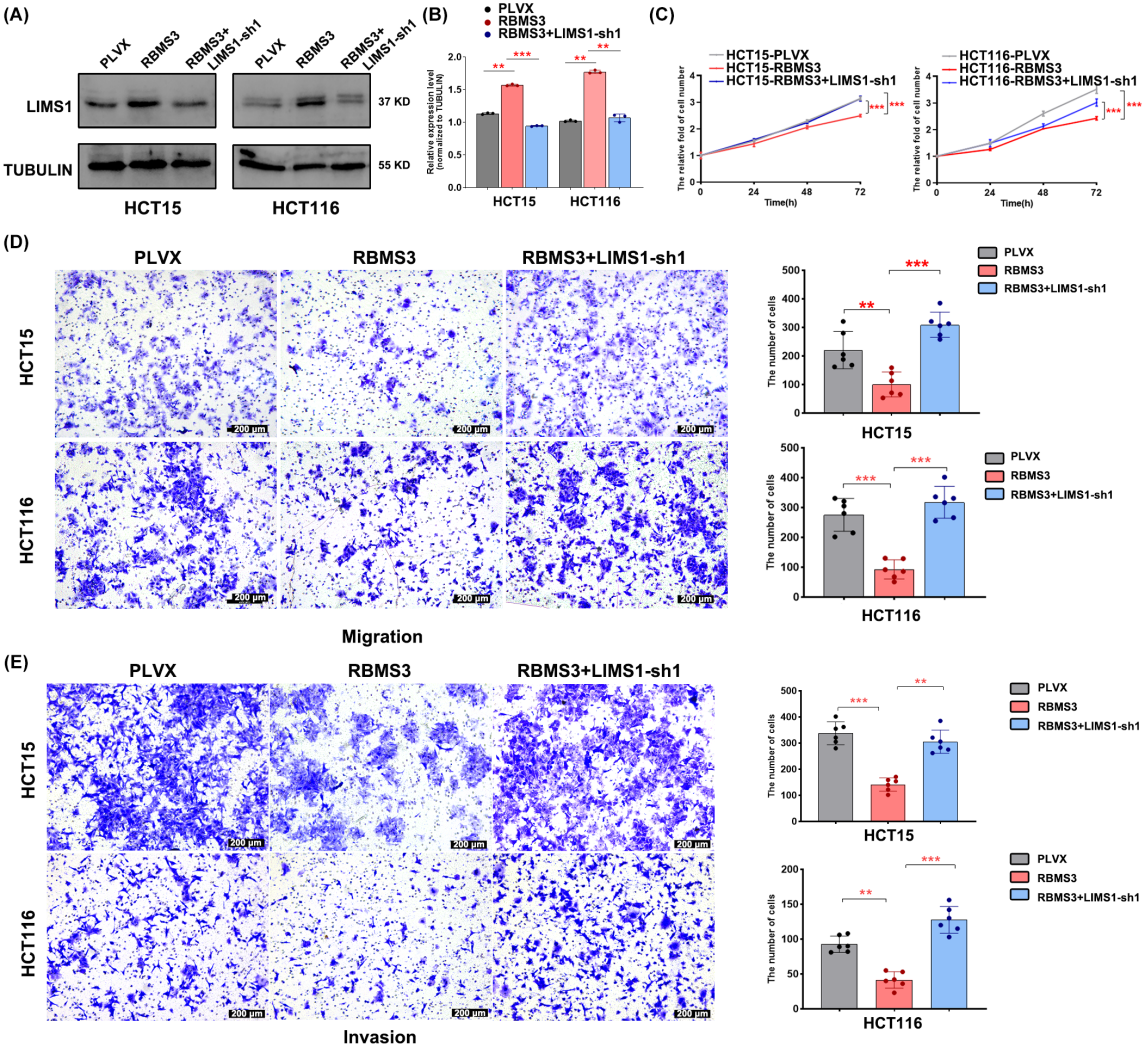
Downregulated LIMS1 recovers the effect of RBMS3 overexpression in colon cancer. (A) Western blot analysis was conducted to evaluate the expression level of LIMS1 in PLVX, RBMS3, and RBMS3+shLIMS1 cell models. (B) The grayscale intensities of the Western blot bands were statistically analyzed, **p < 0.01, ***p < 0.001. (C) Rescued effects of LIMS1 on cell proliferation of HCT15 and HCT116 cells upon RBMS3 overexpression were evaluated. All cells were harvested for MTT analyses at the indicated time points. Data are presented as mean ± SD, ***p < 0.001. (D, E) Rescued effects of LIMS1 on migration and invasion of HCT15 and HCT116 cells upon RBMS3 overexpression were evaluated (magnification, 100×; scale bars, 200 μm). Data are presented as means ± SD, **p < 0.01, ***p < 0.001.

Furthermore, we proceeded to undertake RBMS3 overexpression in HCT116 and HCT15 cells while simultaneously inducing LIMS1 knockdown (Figure 6A,B). Subsequently, we conducted MTT and Transwell migration experiments to investigate whether the downregulation of LIMS1 could counteract the inhibitory effects induced by RBMS3 overexpression on colon cancer cells. Our findings manifest that the knockdown of LIMS1 could indeed rescue cell proliferation, migration, and invasion that were otherwise suppressed by RBMS3 overexpression (Figure 6C–E).

## 4 DISCUSSION

Colon cancer is a common malignant tumor that affects the digestive system. Its underlying mechanisms have been extensively studied. Our research has focused on the role of RBMS3, a gene from the RBM family, in colon cancer. Through differential expression and clinical stage correlation analysis using data from the TCGA and GTEx public databases, we have found that RBMS3 may play a crucial role in colon cancer. Previous studies have reported on the tumor‐suppressive functions of RBMS3 in various cancers, such as breast cancer, esophageal squamous cell carcinoma, and nasopharyngeal cancer.^4^, ^6^, ^16^ Moreover, low expression of RBMS3 has been associated with poor prognosis in cancer patients.^7^, ^17^ However, the role of RBMS3 in colon cancer has not been extensively investigated. In our study, we have observed significant downregulation of RBMS3 in colon cancer tissues compared to normal tissues, as confirmed by IHC, Western blot, and qRT‐PCR. In vitro and in vivo experiments have further demonstrated that upregulation of RBMS3 expression can restrict the cell proliferation, migration, and invasion of colon cancer cells. Importantly, the downregulation of RBMS3 expression has been linked to enhanced growth and metastasis of colon cancer, implying that RBMS3 acts as an inhibitor in colon cancer development.

Previous studies have revealed the regulatory role of RBMS3 in deactivating the Wnt/β‐catenin signaling pathway and repressing the expressions of diverse genes, including β‐catenin, cyclin D1, and C‐myc, within breast cancer cells.^7^, ^18^, ^19^ Nevertheless, the precise molecular mechanisms by which RBMS3 operates in colon cancer remain enigmatic. Our investigation has successfully identified LIMS1 as a prospective target gene of RBMS3 via RNA‐seq and comprehensive database analysis. The utilization of the double luciferase reporter gene assay and RIP assay has confirmed the capacity of RBMS3 to target LIMS1. Furthermore, our study has ascertained that alterations in RBMS3 expression have the ability to impact the stability of LIMS1 mRNA stability, thereby signifying LIMS1's pivotal role as a target gene of RBMS3 in colon cancer.

Originally recognized as a marker for senescent red blood cells, LIMS1 is an evolutionarily conserved protein that exhibits widespread expression within mammalian cells.^20^ It predominantly comprises five zinc finger domains, facilitating its interaction with Integrin‐linked kinase (ILK) and enabling it to function as an adaptor protein for signal transduction in integrin and growth factor pathways.^21^, ^22^ Prior investigations have demonstrated that the association between LIMS1 and RAS suppressor protein 1 (RSU‐1) forms a synergistic complex that effectively inhibits the proliferation and migration of tumor cells.^23^ Intriguingly, the knockout of LIMS1 in hepatic cells leads to liver enlargement and tumor development.^23^ Our research has unequivocally demonstrated that increased expression of LIMS1 impedes the progression of colon cancer, whereas LIMS1 knockdown yields the opposite effect. Notably, LIMS1 has exhibited substantial upregulation in tumor‐associated stromal cells of certain cancers, such as breast carcinomatous tissue, implying that heightened LIMS1 in stroma cells might facilitate cancer progression.^24^ Consequently, our findings propose that LIMS1 might fulfill distinct molecular roles in colon cancer cells versus stromal cells. Furthermore, our study has unveiled the mechanistic involvement of RBMS3, acting as a tumor suppressor, in regulating the mRNA stability of LIMS1, thereby influencing its expression level and consequently restoring LIMS1's inhibitory impact on colon cancer cells. Nonetheless, additional investigations are imperative to the potential role of LIMS1 in stromal cell tumorigenesis and its underlying molecular mechanism.

## 5 CONCLUSIONS

In summary, the downregulation of RMBS3 in colon cancer has been noted. Serving as a potential inhibitor of colon cancer cell proliferation, migration, and invasion, RBMS3 modulates the mRNA stability of LIMS1, thereby exerting a suppressive influence on the advancement of colon cancer.

## AUTHOR CONTRIBUTIONS

**Yafei Li:** Conceptualization (equal); validation (equal); visualization (equal); writing – original draft (equal). **Shuoshuo Wang:** Data curation (equal); formal analysis (equal); software (equal); visualization (equal); Funding acquisition(equal). **Guoli Li:** Resources (equal). **Chunyang Gao:** Funding acquisition (equal). **Zihan Cui:** Investigation (equal). **Mingqi Cong:** Methodology (equal). **Jie Hu:** Resources (equal). **Minghui Zhang:** Funding acquisition (equal). **Xiaoming Jin:** Supervision (equal). **Haiying Sun:** Conceptualization (equal); project administration (equal). **Dan Kong:** Writing – review and editing (equal).

## FUNDING INFORMATION

This work was supported by grants from Innovative Scientific Research Project of Harbin Medical University (No. 31041220040), the Inner Mongolia Science and Technology Plan Project (No. 2020GG0297), the Natural Science Foundation of Inner Mongolia (No.2020MS08084), Postdoctoral Fellowship Program of China Postdoctoral Science Foundation (No. GZC20230640), and the China Postdoctoral Science Foundation (No. 2023M740946).

## CONFLICT OF INTEREST STATEMENT

The authors have no relevant financial or non‐financial interests to disclose.

## ETHICS STATEMENT

Ethical approval was obtained from the Harbin Medical University. This study was reviewed and approved by the Animal Care and Use Committee of Harbin Medical University.

## CONSENT FOR PUBLICATION

All authors have read and agreed to the published version of the manuscript.

## Supporting information


**Figure S1.**.

**Figure S2.**.

**Figure S3.**.

**Figure S4.**.

**Table S1.**.

## Data Availability

Common data for analysis were derived from the following resources available in the public domain: UCSC Xena (https://xenabrowser.net/hub/).
