# Supplementary figures and images for: The RNA‐binding protein RBMS3 inhibits the progression of colon cancer by regulating the stability of LIMS1 mRNA
Source: Cancer Med. 2024 Apr 15;13(7):e7129. doi: 10.1002/cam4.7129 (PMC11017296; doi:10.1002/cam4.7129)

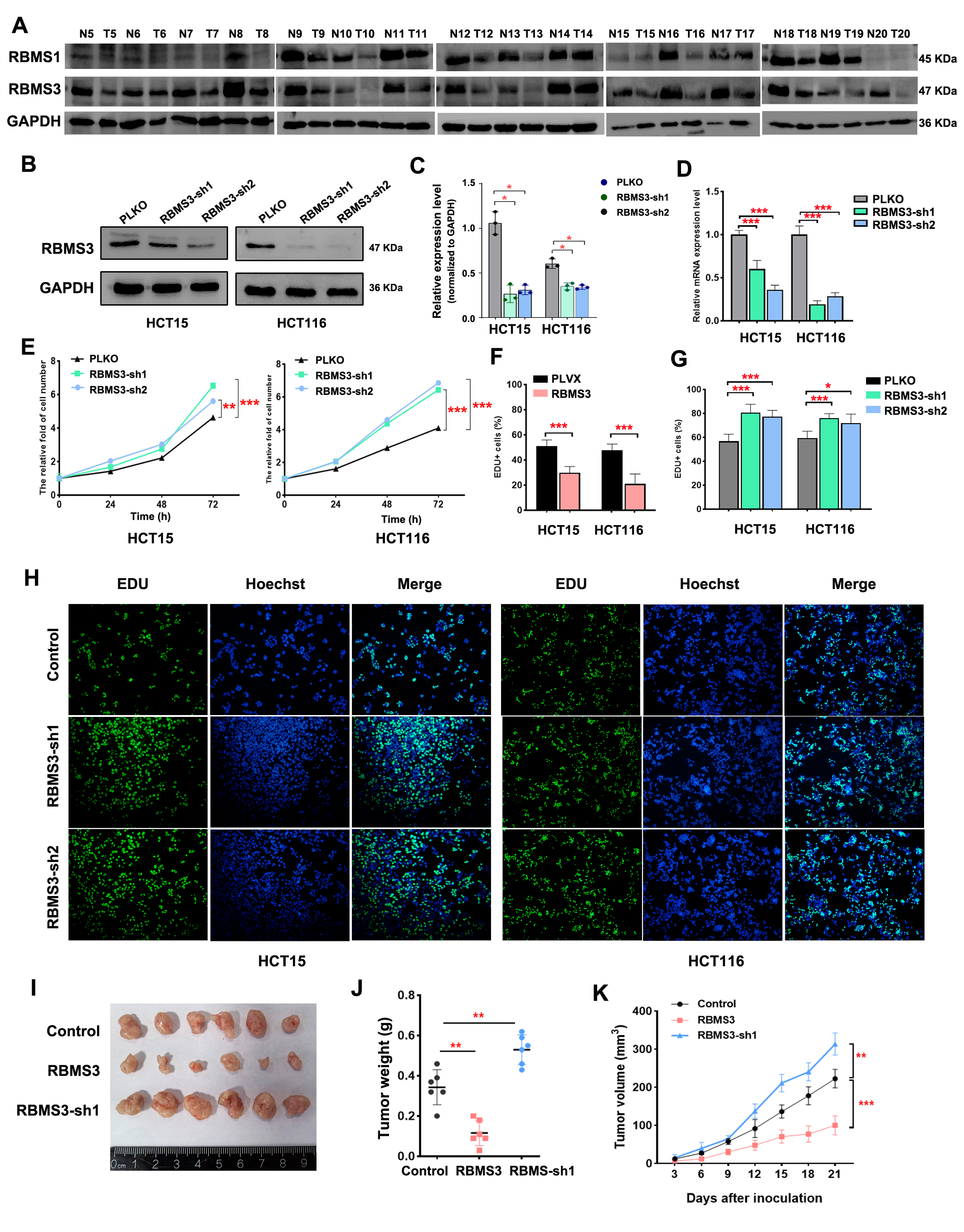

Supplement: Supplementary file 1 — Figure S1.. [file CAM4-13-e7129-s004.tif]

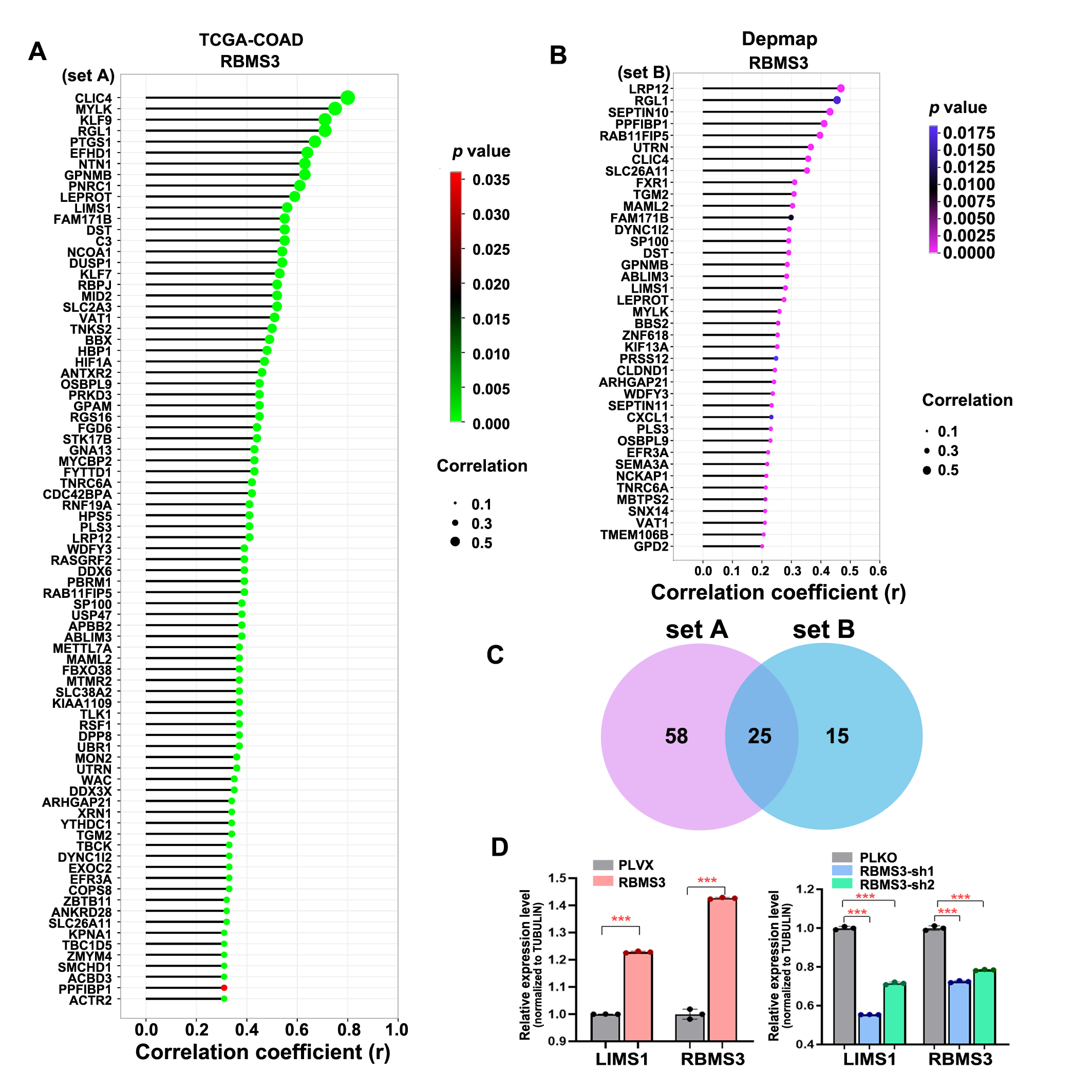

Supplement: Supplementary file 2 — Figure S2.. [file CAM4-13-e7129-s005.tif]

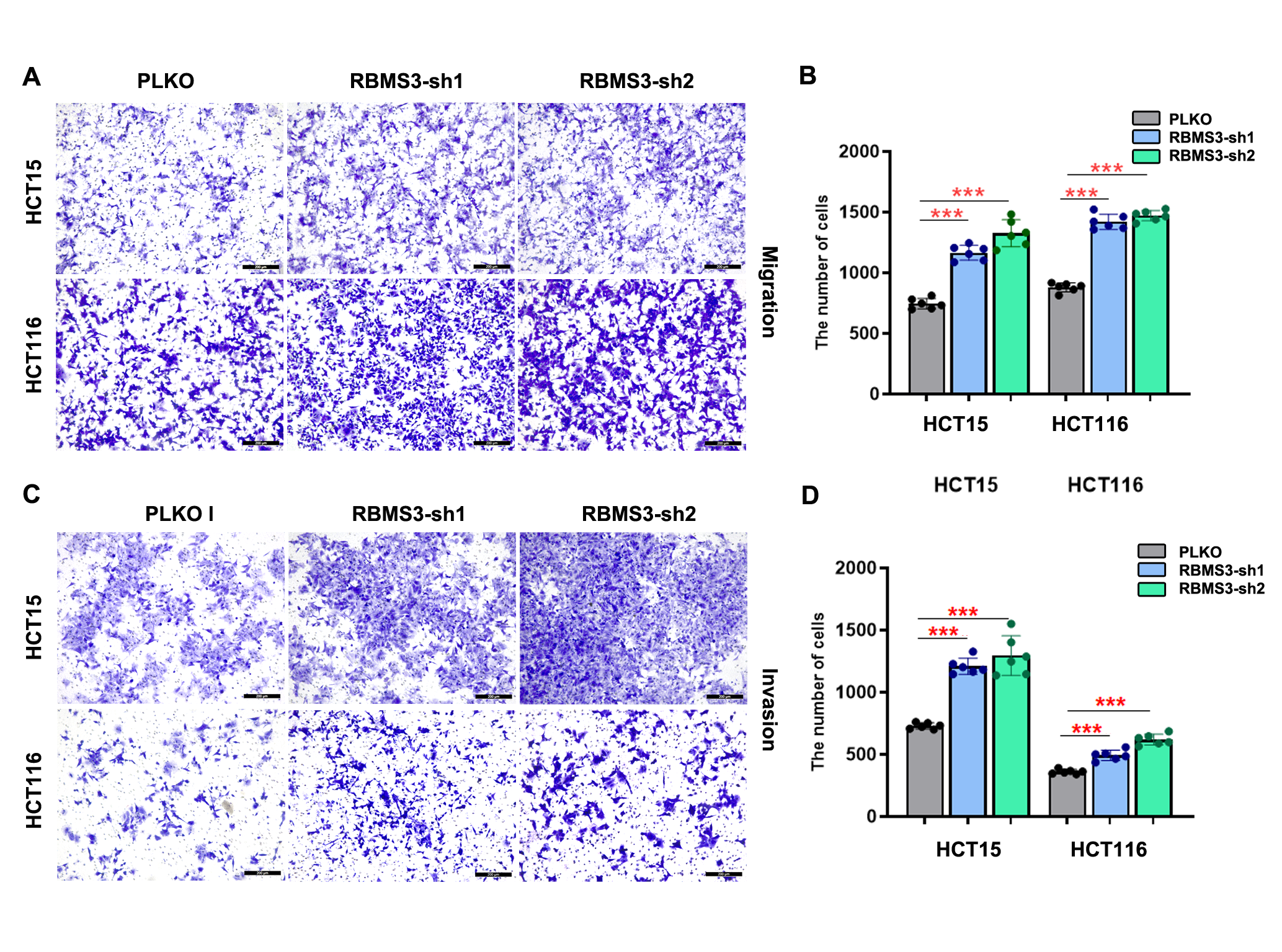

Supplement: Supplementary file 3 — Figure S3.. [file CAM4-13-e7129-s002.tif]

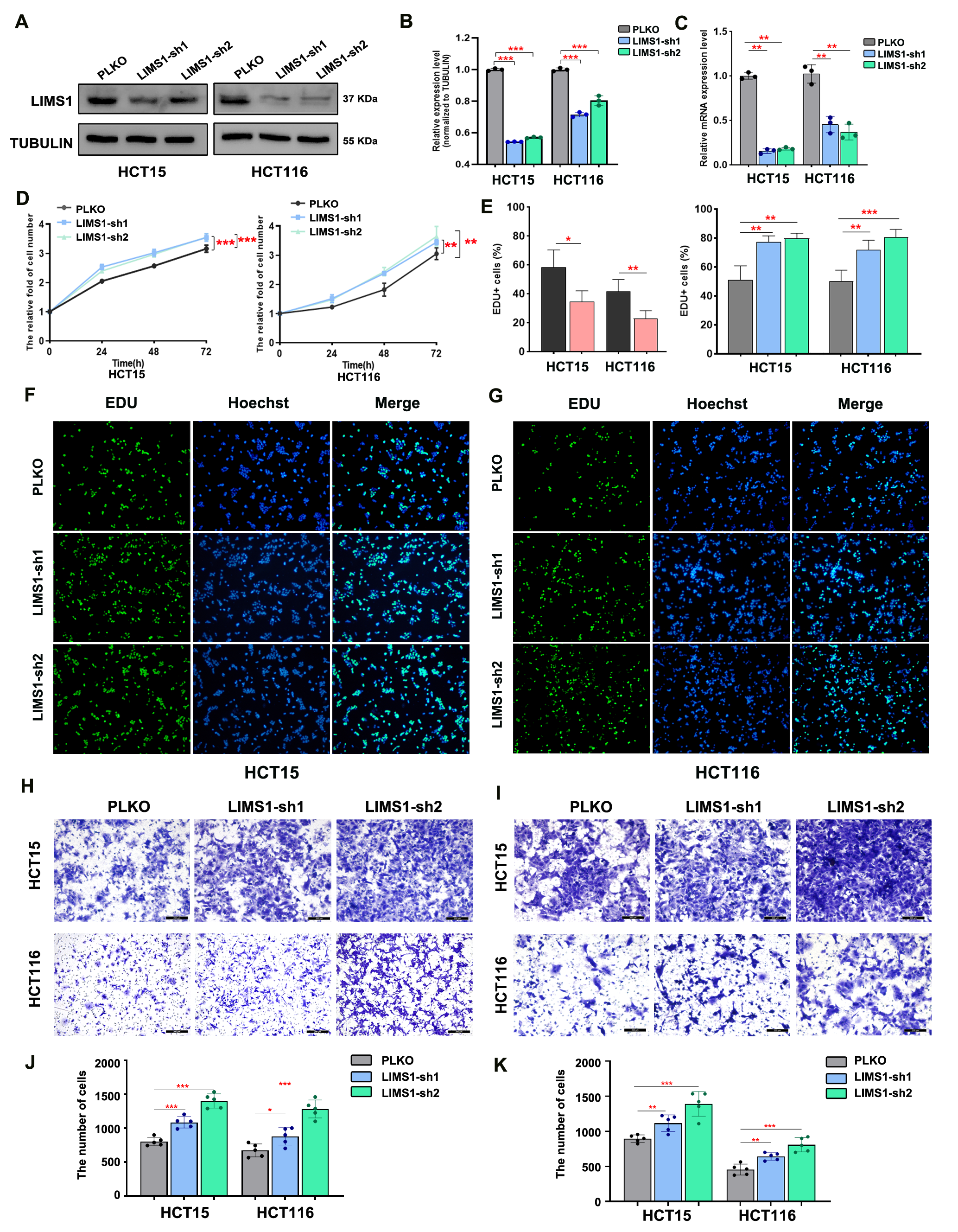

Supplement: Supplementary file 4 — Figure S4.. [file CAM4-13-e7129-s003.tif]
